# Sargramostim for Prophylactic Management of Gastrointestinal Immune-Related Adverse Events of Immune Checkpoint Inhibitor Therapy for Cancer

**DOI:** 10.3390/cancers16030501

**Published:** 2024-01-24

**Authors:** Michael Dougan, Long H. Nguyen, Elizabeth I. Buchbinder, Hillard M. Lazarus

**Affiliations:** 1 Department of Medicine, Harvard Medical School, Boston, MA 02115, USA; michael_dougan@dfci.harvard.edu (M.D.); elizabeth_buchbinder@dfci.harvard.edu (E.I.B.); 2 Department of Medical Oncology, Dana-Farber Cancer Institute, Boston, MA 02215, USA; 3 Division of Gastroenterology, Department of Medicine, Massachusetts General Hospital, Boston, MA 02114, USA; lnguyen24@mgh.harvard.edu; 4 Clinical and Translational Epidemiology Unit, Massachusetts General Hospital and Harvard Medical School, Boston, MA 02114, USA; 5 Department of Medicine, Brigham and Women’s Hospital, Boston, MA 02115, USA; 6 Department of Medicine, Division of Hematology and Oncology, Case Western Reserve University, Cleveland, OH 44106, USA

**Keywords:** sargramostim, granulocyte-macrophage colony-stimulating factor, immune-related adverse events, immune checkpoint inhibitor, cancer, gastrointestinal, colitis, anti-CTLA-4 inhibitor, anti-PD-1/PDL-1 inhibitor, wound healing

## Abstract

**Simple Summary:**

Recently, cancer prognoses have improved by using a new class of drugs called immune checkpoint inhibitors. These treatments work by helping a person’s immune system better recognize and kill cancer cells. Unfortunately, these drugs may cause the immune system to attack normal (i.e., non-cancerous) tissues and can cause serious organ injury. One common and severe toxicity is colitis—inflammation of the bowels—leading to diarrhea, bleeding, and, rarely, death. Sargramostim (recombinant human granulocyte-macrophage colony-stimulating factor (GM-CSF)) has been used clinically for nearly 30 years and has been used as a treatment adjunct for other inflammatory diseases of the bowel, such as Crohn’s disease or ulcerative colitis. Some data suggest that it may be an effective treatment in preventing the side effects of immune checkpoint inhibitor therapy by speeding up the maturation of different blood cells and may quiet inflammation and improve healing in the gastrointestinal tract. Sargramostim may even be linked to prolonged patient survival.

**Abstract:**

Immune checkpoint inhibitor (ICI) therapy improves outcomes in several cancers. Unfortunately, many patients experience grade 3–4 treatment-related adverse events, including gastrointestinal (GI) toxicities which are common. These GI immune-related adverse events (irAEs) induced by ICIs present significant clinical challenges, require prompt intervention, and result in treatment delays or discontinuations. The treatment for these potentially severe and even fatal GI irAEs which include enterocolitis, severe diarrhea, and hepatitis may interfere with the anti-cancer approach. Sargramostim (glycosylated, yeast-derived, recombinant human GM-CSF) is an agent that has been used in clinical practice for more than 30 years with a well-recognized safety profile and has been studied in many therapeutic areas. The mechanism of action of sargramostim may treat moderate-to-severe GI irAEs without impairing the anti-cancer therapy. Some early data also suggest a potential survival benefit. Through the differentiation/maturation of monocytes, macrophages, and neutrophils and induction of anti-inflammatory T cell responses, GM-CSF aids in GI homeostasis, mucosal healing, and mucosal immunity. GM-CSF knockout mice are susceptible to severe colitis which was prevented with murine GM-CSF administration. For some patients with GI mucosa and immune cell function impairment, e.g., Crohn’s disease, sargramostim reduces disease severity. In a prospective, randomized study (ECOG 1608), advanced melanoma patients had a reduction in grade 3–5 GI irAEs and less frequent colonic perforation in the sargramostim plus ipilimumab arm compared to ipilimumab alone. Sargramostim continues to be studied with ICIs for the prophylactic management of irAEs while also potentially providing a survival benefit.

## 1. Introduction

Immune checkpoint inhibitors (ICIs), particularly those targeting programmed death-1/ligand-1 (PD-1/PD-L1) and cytotoxic T lymphocyte antigen-4 (CTLA-4), harness the body’s own defenses to target and destroy tumor cells, revolutionizing cancer care and offering renewed hope to patients with previously untreatable malignancies [1,2]. While these transformative therapies have greatly improved oncologic care, some patients may not achieve the desired anti-tumor responses. Further, ICI therapy may cause immune-related adverse events (irAEs) that require treatment interruptions, dose reductions, or co-administration of medications which themselves may not be well-tolerated or may decrease anti-cancer activity; the treatment may even have to be discontinued entirely [3,4,5,6].

Given the vast surface area of the gastrointestinal (GI) tract [7], its high epithelial turnover [8], and its established role as a site for immune surveillance of foreign antigens (e.g., the microbiome, diet, other xenobiotics, etc.) that can become maladapted in the context of autoimmunity [9,10], it is not surprising GI irAEs due to ICI therapy—including enterocolitis and hepatitis—are common, morbid, and in some cases, fatal [4,11].

The development of strategies to alter this biology may offer the promise of amplifying the potency and broadening the use of checkpoint blockade in cancer therapy by improving the safety profile of these revolutionary medications. Prophylactic medications to prevent or ameliorate GI irAEs may enable the benefits of ICIs to be extended to a greater number of patients.

We review our current understanding of GI irAEs due to ICI therapy and discuss rescue treatments for mild, moderate, and severe toxicity. Unfortunately, these approaches may impair the anti-cancer response. We then present emerging data for the use of sargramostim (glycosylated, yeast-derived, recombinant human granulocyte-macrophage colony-stimulating factor (rhu GM-CSF), Leukine^®^) to prevent GI irAEs. Sargramostim is an agent that has been used in clinical practice for more than 30 years and has a well-recognized safety profile with therapeutic potential as a host-directed immunotherapy. Its unique mechanism of action may enable the management of moderate-to-severe GI irAEs without impairing anti-cancer therapy. We review and present literature that may support the use of sargramostim as a viable therapeutic adjunct.

## 2. Gastrointestinal Immune-Related Adverse Events

Checkpoint blockade, similar to other immunotherapies (e.g., adoptive T cell or cytokine-based), has the potential to induce diverse systemic toxicities [6,11], manifesting as either single organ injury or multiple system toxicities [12]. These toxicities often occur weeks to months after treatment initiation [13]. While irAEs have been reported involving nearly every organ system (e.g., neurologic, cardiac, and endocrine), ICI toxicities most often affect barrier organs, such as the skin, liver, and luminal GI tract, likely owing to the immune regulatory role for PD-1/PD-L1 and, particularly, CTLA-4 at these respective sites [12,14]. Correspondingly, CTLA-4 inhibitors (e.g., ipilimumab and tremelimumab)—alone or in combination—are more frequently associated with GI irAEs, such as enterocolitis or hepatitis, as opposed to PD-1/PD-L1 blockade alone [6,13,14]. The management of ICI irAEs has been discussed extensively elsewhere (“Management of Immune-Related Adverse Events in Patients Treated With Immune Checkpoint Inhibitor Therapy: ASCO Guideline Update”) [15].

GI irAEs most commonly involve the colon with approximately 80% of patients with GI irAEs showing involvement on histopathology, although a substantial subset have inflammation isolated to the stomach or small intestine (~20%) [16,17]. Although most GI irAEs are relatively mild, 15–20% of patients receiving CTLA-4 inhibitors and 2–5% of patients receiving PD-1/PD-L1 inhibitors will develop severe enterocolitis, with a smaller fraction progressing to life-threatening inflammation (Table 1) [14,18,19]. Best practices for the diagnosis and treatment of GI irAEs have also been discussed elsewhere and are beyond the scope of this review (“AGA Clinical Practice Update on Diagnosis and Management of Immune Checkpoint Inhibitor Colitis and Hepatitis: Expert Review”) [19].

Mild GI irAEs are generally managed with empiric, symptom-directed treatment [19]. Patients experiencing symptoms that affect daily living should undergo diagnostic testing. The pattern of GI luminal involvement and severity of mucosal injury observed on endoscopy correlate poorly with both symptoms and cross-sectional imaging [18,20]. Consequently, endoscopy plays a critical role in the management of these toxicities [19]. The management of GI irAEs should be tailored to the results of testing and may include withholding ICI therapy and treating with immunosuppression. See Section 2.3 below.

### 2.1. Mechanisms Underlying GI irAEs

**Figure 1:**
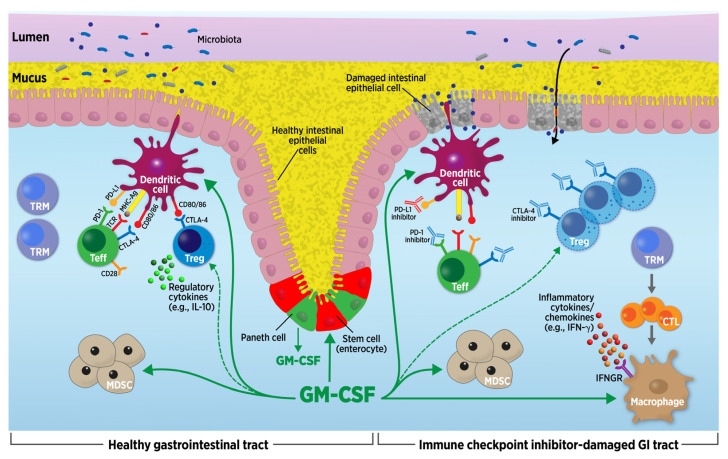
GM-CSF interactions in the healthy and immune checkpoint inhibitor (ICI)-damaged gastrointestinal (GI) tract. Healthy GI tract depicted on the left. GI tract with ICI-related toxicities depicted on the right. ICI GI toxicities are caused by inflammatory pathways after CTLA-4 and/or PD-1 inhibition. Endogenous granulocyte-macrophage colony-stimulating factor (GM-CSF) is secreted throughout the body by many cells including the GI tract Paneth cells. GM-CSF may enhance Paneth cell immune function (e.g., CD80 and CD86 expression). GM-CSF enhances GI crypt cell/enterocyte proliferative activity, contributing to intestinal epithelial cell homeostasis. GM-CSF affects myeloid-derived cells, including macrophages, dendritic cells, myeloid-derived suppressor cells (MDSCs), and regulatory T cells (Tregs) (indirectly, i.e., dashed line). Sargramostim (recombinant human GM-CSF) administration should have the same effect as endogenous GM-CSF. In ICI-damaged GI tracts, immune infiltrates consist predominately of activated effector CD8+ T cells (Teff). Tissue-resident memory T cells (TRM) differentiate into cytotoxic T lymphocytes (CTL) which stimulate inflammatory cytokine production. Regulatory (anti-inflammatory) cytokines (e.g., IL-10) are diminished. Myeloid-derived cells (e.g., macrophages/monocytes, dendritic cells) adopt an inflammatory phenotype that may occur in response to interferon-gamma (IFN-γ) exposure. Tregs expand but are suppressed by CTLA-4 blockade. Microbiota penetrate the intestinal barrier. GM-CSF may decrease inflammation through activation of monocyte-derived MDSCs. Figure modified from Tang et al. (2021) (Ref. [24]) and Luoma et al. (2020) (Ref. [21]). Abbreviations: CTL = cytotoxic T lymphocyte; GI = gastrointestinal; GM-CSF = granulocyte-macrophage colony-stimulating factor; IFN-γ = interferon-gamma; IFNGR = interferon-gamma receptor; MDSC = myeloid-derived suppressor cell; Teff = effector T cell; Treg = regulatory T cell; TRM = tissue-resident memory T cell.

The mechanisms driving GI irAEs are beginning to be understood (Figure 1) [12,21,22]. These varied processes may lead to severe GI symptoms in the patient. As with most irAEs visualized on biopsy, GI luminal toxicities demonstrate an acute lymphocytic and neutrophilic response [13,23]. Signs of chronic structural changes to the colon are generally absent. Detailed analyses of this immune infiltrate have indicated that these cells are predominately activated effector CD8+ T cells (Teff) that are proliferative and express high levels of granzyme B and interferon-gamma (IFN-γ) [21,22]. Perhaps not surprisingly, these cells also highly express PD-1 and CTLA-4, along with a number of other immune checkpoint proteins (Figure 1) [21]. A clonotypic analysis of these cells indicated that they originate from the resident memory T cell (TRM) pool in the colonic mucosa. Tang et al. [24] suggest that a proinflammatory state results due to the hyperactivation of Teff cells and differentiation of CD8+ TRMs to cytotoxic T lymphocytes (Figure 1). A smaller expansion of CD4+ T cells with an IFN-γ-secreting TH1 phenotype is also evident, while other helper T cell populations appear to be unchanged (e.g., TH17) [21]. These cells do not appear to be related to TRMs, suggesting that they originate outside the colon. In contrast to early predictions, regulatory T cells (Tregs) are abundant in the inflamed colons of patients with luminal GI irAEs, although the production of anti-inflammatory cytokines like IL-10 is reduced (Figure 1) [6,12,21]. While Tregs expand, the cells are suppressed by the CTLA-4 blockade (Figure 1) [21]. Myeloid-derived cells (e.g., macrophages/monocytes, dendritic cells) also play a central role in GI luminal toxicities, adopting an inflammatory phenotype that may occur in response to exposure to IFN-γ (Figure 1). These myeloid cells produce and react to tumor necrosis factor (TNF)-alpha, a cytokine that likely underlies much of the pathology in the disease [12,21]. B cells appear phenotypically normal, and, as of yet, do not seem to have any role in GI irAEs [21,22]. In a healthy GI tract, the gut microbiome also plays a critical role in the regulation of intestinal mucosal homeostasis (Figure 1) [25,26]. Tang and colleagues [24] proposed that immunotherapy modulates and disrupts the microbiota–gut barrier (Figure 1).

Why some patients develop these GI irAEs and others do not remains unclear, particularly since TRMs are universally present throughout the gut [12]. Understanding the factors that contribute to this risk may provide insights that could lead to more tailored interventions, particularly those aimed at preventing luminal toxicities.

### 2.2. Lessons Learned about GI irAEs from Inflammatory Bowel Disease

**Figure 2:**
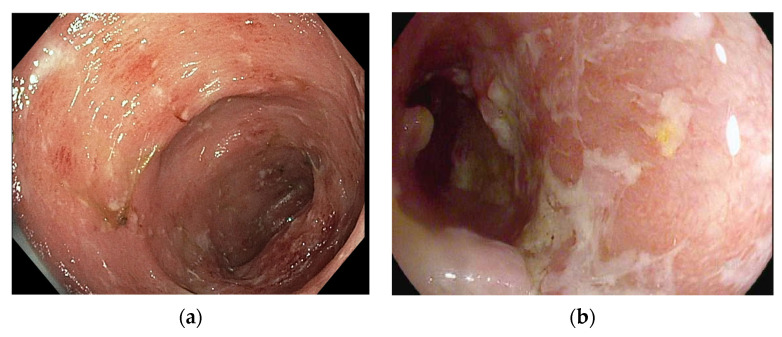
Gastrointestinal immune-related adverse event (GI irAE) colitis resembles colitis due to inflammatory bowel disease (IBD) which includes ulcerative colitis and Crohn’s disease. High-resolution endoscopic photographs using a standard lens showing colitis (**a**) from a patient with melanoma receiving ipilimumab and nivolumab and (**b**) a patient with ulcerative colitis. The findings in both show an edematous, granular, and erythematous mucosa with scattered erosions and excess mucus. In (**b**), an inflammatory polyp is also visible.

Immune checkpoint inhibitor-induced GI luminal toxicities share several features with inflammatory bowel disease (IBD), a spontaneous inflammatory disease of the GI tract which is split into two subtypes, ulcerative colitis and Crohn’s disease (Figure 2) (Table 2) [14,23]. The symptoms of GI irAEs overlap with all forms of colitis, including IBD and infectious colitis; these symptoms include urgency, cramping, frequent watery bowel movements, and, less commonly, abdominal pain and bloody diarrhea [14,19]. Many patients with toxicities that result from ICIs also have nausea and vomiting, which are less frequent in IBD but are still common.

Similar to IBD, morphological changes on endoscopy associated with GI irAEs include edema, erythema, erosions, spontaneous bleeding, and occasionally, deep ulcerations (Figure 2 and Table 2) [18,20,23]. In most patients, this inflammation extends throughout the entire colon, a characteristic more similar to ulcerative colitis than to Crohn’s disease which often appears in distinct patches or “skip lesions” [14,23,27]. Strictures, another feature of Crohn’s disease, are rarely observed in the GI luminal toxicities from checkpoint blockade [14]. On biopsy, GI irAEs are difficult to distinguish from IBD, although IBD commonly shows evidence of chronic changes to the colonic mucosa that are not observed in checkpoint blockade toxicities [23].

Although checkpoint blockade GI irAEs are clearly a distinct syndrome from IBD, some mechanistic overlap likely exists (Table 2). In addition to similarities in appearance and on biopsy, patients with IBD also have a substantially higher risk for developing GI luminal toxicities from checkpoint blockade than patients without IBD [28]. In a multicenter retrospective study of more than 100 patients with IBD treated with checkpoint blockade, 41% developed a GI luminal toxicity compared to 11% of those who did not have IBD [28]. This risk included 21% of patients who developed severe GI toxicities including several patients with perforations.

### 2.3. Treatment of GI irAEs

Because of the similarities to IBD, the initial strategies to treat GI irAEs have all come from the standard therapies for IBD [18,19]. Currently, there are no randomized controlled trials of therapies for GI luminal toxicities from checkpoint blockade, and all guidelines are based on retrospective analyses or prospective trials with small numbers of patients [19]. The first-line treatment remains systemic glucocorticoids (Table 3), which are effective in approximately two-thirds of patients. For those who do not respond adequately to glucocorticoids, TNF-alpha inhibitors, such as infliximab, and the integrin inhibitor vedolizumab have shown evidence of efficacy, leading to a resolution of the inflammation in most patients (Table 3) [19,29,30]. These classes of drugs are standard first-line maintenance therapies for moderate-to-severe IBD [31,32]. Similarly, for highly refractory cases of GI luminal toxicities, two additional therapies approved for IBD, the IL-23p40 inhibitor ustekinumab and the Janus kinase (JAK) inhibitor tofacitinib, appear to be effective, although the data supporting their use are more limited (Table 3) [33,34].

A single randomized controlled trial examined a colon-targeted formulation of the glucocorticosteroid budesonide as a prophylaxis for checkpoint blockade colitis [35]. This trial was negative, demonstrating no benefit to pretreating patients prior to the onset of colitis symptoms. At this point, no specific therapies are recommended for preventing GI irAEs, although such therapies are urgently needed clinically.

## 3. Development and Biologic Effects of Granulocyte-Macrophage Colony-Stimulating Factor (GM-CSF)

**Figure 3:**
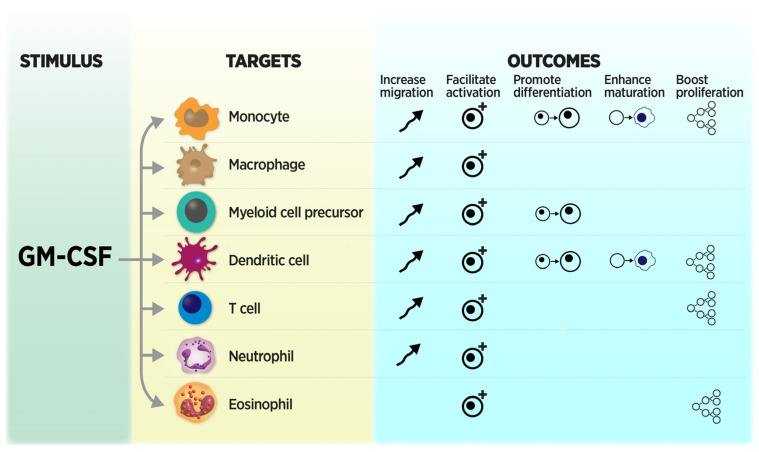
GM-CSF, a pleiotropic cytokine, has diverse effects on many marrow-derived and blood cells. The resulting effects are the increased migration, activation, differentiation, maturation, and proliferation of many effector cells. Note: The effects of GM-CSF on T cells are indirect. GM-CSF also influences non-immune cells including epithelial cells such as enterocytes. Symbols depict the indicated outcome. Figure modified with author permission from Damiani et al. (2020) (Ref. [41]).

GM-CSF was initially identified in the 1960s as a myeloid growth factor, and was ultimately purified in the 1970s. GM-CSF underwent molecular cloning in the 1980s and entered the clinical therapeutic arena in the 1990s [36,37,38,39]. At first, GM-CSF was recognized for its effects on hematopoiesis, particularly the myeloid lineage, that included the proliferation, activation, maturation, and differentiation of committed hematopoietic progenitors (Figure 3) [36,40]. Subsequently, GM-CSF has been demonstrated to be an immunomodulatory cytokine that can have more widespread effects [36,41,42,43,44]. GM-CSF is mainly produced by T helper (CD4^+^) lymphocytes and innate lymphoid cells; the non-hematopoietic cell populations, which are able to secrete GM-CSF in response to specific stimuli, include fibroblasts, endothelial cells, and alveolar epithelial cells [45,46]. In vitro, GM-CSF stimulates cellular and humoral immunity through the activation of neutrophils, macrophages, and dendritic cells, and induces the activation of T cells including modulation of the TH1/TH2 cytokine balance [47,48]. Specifically, GM-CSF activates dendritic cells to promote antigen presentation and, thus, enhances T cell antigen-specific responses [49]. These broad pleiotropic effects, coupled with its ability to link the innate and adaptive immune responses through effects on dendritic cells and T cells, highlights the central role of GM-CSF in immune response regulation.

Endogenous GM-CSF plays a much smaller role in steady state homeostatic myelopoiesis when compared to granulocyte colony-stimulating factor (G-CSF), as has been demonstrated in mice carrying a null allele of the GM-CSF gene [50,51,52]. Such mice exhibit steady-state hematopoiesis and a normal granulocyte and monocyte lifespan [46,50]. On the other hand, in inflammatory settings, GM-CSF will dramatically increase monocyte and neutrophil bone marrow production with a marked expansion and differentiation of myeloid cells (Figure 3) [51]. Importantly, the participation of GM-CSF in the host response is essential for resisting local infections. GM-CSF contributes significantly to normal pulmonary physiology [53]. A deficiency in GM-CSF resulting from neutralization by autoantibodies causes the development of the disease known as pulmonary alveolar proteinosis (aPAP). This condition is characterized by a failure of lung alveolar macrophages to clear surfactant, the lipoprotein secreted by lung alveolar cells, as these cells depend on endogenous GM-CSF stimulation. The excess accumulation of surfactant protein in the alveoli impairs the differentiation of monocytes into macrophages, causing a loss of protection and inability to repair damaged epithelial barriers [53,54,55,56]. Decreased peroxisome proliferators-activated receptor γ (PPAR-γ) expression also leads to dysregulated cholesterol clearance in the alveoli [53].

We can infer from the above that the GI tract also exhibits dysfunction in the setting of endogenous GM-CSF deficiency. Based on similar immune cell populations and GM-CSF effects on epithelial barrier cells in inflammatory lung and GI tract disease (e.g., influenza, aPAP, and Crohn’s disease), decreased endogenous GM-CSF leads to unopposed or dysregulated inflammation from reduced Treg and monocytic myeloid-derived suppressor cell (MDSC) numbers that dampen cytokine production, T cell proliferation, and chemotaxis; these effects damage the lungs and GI tract [27,53,57,58,59]. Mucosal repair is diminished and recovery is inhibited, along with decreased induction, recruitment, and survival of dendritic cells [27,58,60]. Additionally, an altered host immune response can increase susceptibility to infection (Figure 1) [61].

GM-CSF secreted by Paneth cells, critical intestinal immune cells in the base of the GI crypts, and hematopoietic cells contribute to intestinal epithelial cell homeostasis via several mechanisms including the maintenance of CD103^+^ dendritic cells (Figure 1) [62,63,64]. GM-CSF may enhance Paneth cell immune function (e.g., CD80 and CD86 expression) [62]. This relationship supports the generation of regulatory T cells which control inflammation, mucosal repair, and immune tolerance [65,66]. In fact, through the differentiation and maturation of monocytes, macrophages, and neutrophils, and induction of anti-inflammatory T cell responses, GM-CSF aids in GI homeostasis, mucosal healing, and mucosal immunity (Figure 1 and Figure 3) [62,63,67,68,69]. GM-CSF may also decrease inflammation through the activation of MDSCs (Figure 1) [68].

In a preclinical study, Dranoff and colleagues [70] showed that irradiated tumor cells engineered to express murine GM-CSF stimulated potent, long-lasting, and specific anti-tumor immunity. Intralesional use of GM-CSF resulted in an increase in tumor-infiltrating CD4^+^ T cells that enhanced CD8+ T cells and increased the number of tissue-resident Langerhans macrophages in the skin [71]. In several tumor types, the macrophage response may result in increased phagocytosis of tumor cells and the recruitment of dendritic cells to the tumor and sentinel lymph nodes [72,73]. GM-CSF also stimulates tumor-infiltrating macrophages to produce more angiostatin which suppresses angiogenesis, a critical component in the metastatic cascade [74]. Within the GI tract, these effects may lead to enhanced anti-tumor T cell priming and activation via increased dendritic cell tumor-associated antigen presentation [36,75].

### 3.1. Recombinant GM-CSF Expression Systems

While three formulations of rhu GM-CSF have been studied, they differ in their glycosylation based on the expression systems in which they are produced and, thus, are not interchangeable [76,77]. Glycosylation is a significant biologic feature that influences the pharmacokinetics, activity, and safety profile of each drug formulation [76,78]. Neither molgramostim (produced by prokaryotic *Escherichia coli* (bacteria) and is not glycosylated) nor regramostim (mammalian-derived from Chinese hamster ovary cells and is glycosylated) are commercially available [76,77]. The third product, sargramostim, is yeast-derived and possesses a glycosylation pattern similar to native GM-CSF; this property conveys biologic activity, stability, resistance to degradation, tolerability, toxicity, and immunogenicity similar to native GM-CSF. In light of its ability to increase immune cell proliferation, maturation, and activation, and enhance tumor antigen presentation by dendritic cells, sargramostim has been widely studied as an adjunct to cancer therapy.

### 3.2. Gastrointestinal Therapeutic Uses of Recombinant GM-CSF

#### 3.2.1. Pre-Clinical Studies

The data regarding endogenous GM-CSF strongly suggest that exogenous administration may have a number of potential therapeutic uses. Preclinical studies have shown protective properties of GM-CSF on the GI tract. GM-CSF-knockout mice are susceptible to colitis that was prevented with murine GM-CSF administration [69,79,80]. Sainathan et al. [79] also demonstrated that GM-CSF is effective in RAG1(−/−) mice, demonstrating that GM-CSF activity is independent of effects on T and B cells. Sennikov and associates [81] developed a murine in vitro preclinical model using cultured media derived from intestinal crypt cells that was demonstrated to contain GM-CSF. Both the cultured media as well as native GM-CSF enhanced GI crypt cell proliferative activity in vitro. Han and co-workers [82] used a non-steroidal anti-inflammatory drug (NSAID) model in GM-CSF-null mice. Loss of GM-CSF signaling in non-hematopoietic cells increased the degree of NSAID-induced ileal injury indicating that GM-CSF signaling is essential for ileal epithelial homeostasis and homeostatic intestinal barrier function. In work by Egea and colleagues [83] using the dextran sodium sulfate (DSS) colonic epithelial-injury model in GM-CSF-deficient mice, demonstrated that the decreased crypt cell proliferation and delayed ulcer healing in the GM-CSF-deficient mice were rescued by the administration of exogenous GM-CSF. Bernasconi et al. [84] also used a DSS colitis murine model and compared treatment with daily pegylated GM-CSF versus saline. They reported that the GM-CSF therapy reduced the clinical signs of colitis and the release of inflammatory mediators. Histologically, they noted improved mucosal repair, faster ulcer re-epithelialization, an accelerated hyperproliferative response of epithelial cells in ulcer-adjacent crypts, and increased accumulation of CD11b+ monocytic cells in colonic tissues.

Preclinical studies have also investigated wound healing in the GI tract. Intraperitoneal chemotherapy (IPCT) has been used as an alternative local adjuvant treatment for patients with resectable colonic and gastric cancers to deliver higher local concentrations of cytotoxic drugs with diminished systemic toxicity when compared to intravenous chemotherapy. Intraoperative irradiation has also been used to reduce the number of local recurrences and to increase disease-free survival in the treatment of intestinal malignancies [85]. The intestinal anastomotic leakage that may accompany these interventions is a serious surgical complication, and both IPCT given in the early postoperative period as well as intraoperative irradiation adversely affect the healing of intestinal anastomoses. Experimental studies have been conducted in rats in the early postoperative IPCT setting to assess the local administration of recombinant GM-CSF into the perianastomotic area [86,87]. These investigations examined the bursting pressures of anastomotic segments, hydroxyproline contents for levels of anastomotic collagen, and histologic immune and pathology effects. Both Erdem and colleagues [86] and Cetinkaya and colleagues [87] found an impairment in anastomotic healing along with diminished peritoneal macrophage activity after intraperitoneal administration of 5-fluorouracil (5-FU) or mitomycin-C. A local injection of recombinant GM-CSF to both sides of the colonic anastomoses enhanced wound healing and was associated with increased macrophage activity, bursting pressures, and hydroxyproline levels. Similarly, Dinc and colleagues [88] showed in a preclinical intraoperative irradiation rat model that the local injection of recombinant GM-CSF improved the wound healing of intraoperatively irradiated bowel anastomosis. These IPCT and irradiation studies are further supported by clinical evidence in which locally administered GM-CSF was able to successfully treat chronic and incisional dermal wounds [89,90]. These findings suggest that recombinant GM-CSF applied locally may enhance healing and prevent anastomotic leakage, allow for the use of higher doses of postoperative chemotherapeutics, and reduce postsurgical risks in the resection and anastomosis of irradiated intestines.

#### 3.2.2. Clinical Studies

GM-CSF concentrations and off-label use of rhu GM-CSF have been studied in a range of GI-related uses. Brubaker and colleagues [91] reported subjects who had higher serum concentrations of IL-10 and GM-CSF were protected from enterotoxigenic *E. coli* (ETEC) diarrhea in an intestinal and systemic inflammation challenge model using an experimental ETEC strain. Buskirk et al. [92] similarly reported that higher stool concentrations of GM-CSF were associated with lower disease severity and the absence of diarrhea in children with enteropathogenic *Escherichia coli* (EPEC) infection and dysenteric Shigella infection. In a prospective, randomized study of patients with moderate-to-severe active Crohn’s disease (n = 124), Korzenik and colleagues [93] reported that sargramostim (6 µg/kg/day subcutaneously for 56 days) decreased disease severity and improved quality of life compared to a placebo. Meropol et al. [94] concluded that sargramostim treatment may permit an increased 5-FU dose intensity by acting as a GI mucosal protectant in cancer patients treated with weekly IV 5-FU (mean weekly dose intensity of 424 mg/m^2^), high-dose leucovorin, and subcutaneous sargramostim 250 mcg/m^2^ 5 days each week. Melichar and associates [95] found a significant improvement in GI permeability after chemotherapy with oral 200 µg molgramostim mouthwashes (swish and swallow) taken daily for 4 days for chemotherapy-induced stomatitis (n = 10) compared to control patients with cancer (n = 21). Finally, Chachoua and associates [96] demonstrated that sargramostim treatment led to a significant enhancement in direct monocyte-induced cytotoxicity in vitro against human colon adenocarcinoma cell line HT29 cells. In summary, higher GM-CSF concentrations were linked to a lower disease severity and diarrhea in infections that commonly present with high rates of diarrhea [91,92]. Recombinant human GM-CSF administration was reported to decrease Crohn’s disease severity and improve quality of life [93], allow for increased chemotherapy dosing by serving as a GI mucosal protectant [94], improve GI permeability in chemotherapy-induced stomatitis [95], and increase monocyte-induced cytotoxicity of colon adenocarcinoma [96].

These preclinical and clinical data indicate that sargramostim may be a useful option in GI inflammatory diseases. ICI-induced inflammation and immune response dysregulation may damage GM-CSF-producing GI tract tissues in immune-mediated colitis as well as lung tissue in checkpoint-induced pneumonitis [14,97]. The use of rhu GM-CSF may mitigate moderate-to-severe immune-related adverse events (irAEs), including GI irAEs; this approach additionally may provide a survival benefit [42,45].

## 4. Clinical Studies of Sargramostim (rhu GM-CSF) with Immune Checkpoint Inhibitors

Cancer outcomes have been improved with ICI therapies. However, as previously mentioned, auto-immune toxicities associated with ICI therapy (e.g., irAEs) can lead to severe morbidity, treatment discontinuation, and, in rare cases, death. There is a need for combination strategies which improve the responses to immune checkpoint inhibition and/or limit the auto-immune toxicities. One potential therapy to add to immune checkpoint inhibition is sargramostim. Pre-clinical animal models have suggested a benefit to combining immune checkpoint therapy with GM-CSF-secreting whole cell vaccines in controlling melanoma [98]. This pre-clinical experience and the evidence described above led to clinical trials testing rhu GM-CSF with ICI therapy specifically in the cancer setting.

**Figure 4:**
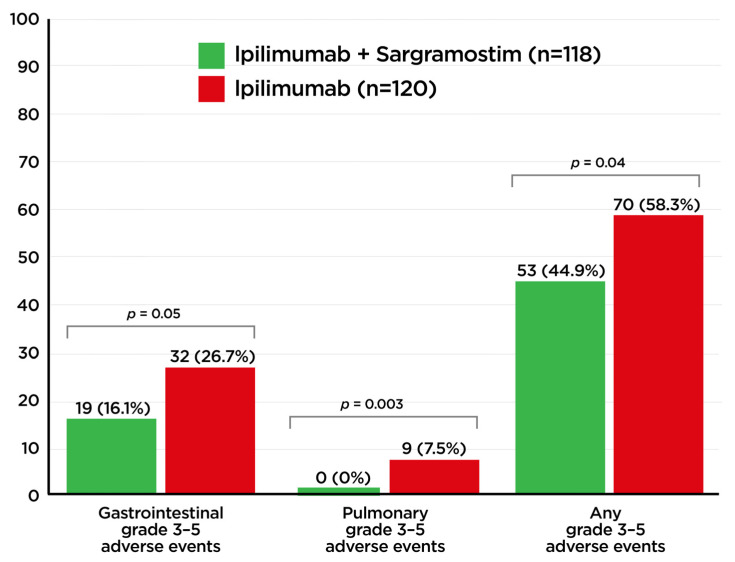
Addition of sargramostim (recombinant human granulocyte-macrophage colony-stimulating factor (rhu GM-CSF)) to ipilimumab significantly lowered grade 3–5 adverse events compared to the ipilimumab-alone arm in a multi-center, randomized clinical trial for advanced melanoma (Hodi 2014) (Ref. [99]).

A multicenter, randomized trial combining CTLA-4 inhibition (ipilimumab) and sargramostim for the treatment of advanced melanoma was performed by Hodi et al. (ECOG 1608) (Table 4) [99]. This study demonstrated that the addition of sargramostim improved the overall survival for the combination therapy compared to the CTLA-4 inhibitor alone (17.5 months versus 12.7 months; *p* = 0.01). However, the progression-free survival for both arms was identical at 3.1 months. The patients treated with the combination of CTLA-4 inhibition and sargramostim had a lower rate of severe toxicity (grade 3–5 irAEs) than those subjects treated with CTLA-4 inhibition alone (44.9% versus 58.3%; *p* = 0.04) (Figure 4). This was particularly observed for GI and pulmonary grade 3–5 irAEs (*p* = 0.05 and 0.003, respectively). Less colonic perforation (1.7% vs. 5.8%) was observed in patients treated with the combination of CTLA-4 inhibition and sargramostim. These data suggest that adding sargramostim to CTLA-4 inhibition might improve overall survival by lowering toxicity which allows patients to remain on therapy longer. Fewer patients in the ipilimumab and sargramostim group (25.6%) discontinued treatment due to AEs compared to the ipilimumab alone group (36.1%). In addition, the lower rates of colonic perforation support the role that GM-CSF plays in protecting against colitis and GI irAEs due to ICI therapy. Additionally, GM-CSF can drive the differentiation of dendritic cells and improve anti-tumor immunity in some settings, although this effect appears to be dose-dependent [36,41,42,43]. It is also possible that part of the positive response of GM-CSF therapy in Crohn’s disease and in ICI-induced enterocolitis is through a better defense against invading microbes and an enhancement of wound healing [42].

Additional melanoma trials have also combined sargramostim with CTLA-4 inhibition (Table 4). These investigations include a prospective, phase 2 clinical trial performed by Kwek et al. [100] using CTLA-4 antibody with sargramostim in 22 metastatic melanoma patients which found similar outcome and safety results as those of the Hodi et al. [99] randomized trial (Table 4). A single-institution, retrospective study of 32 melanoma patients treated using CTLA-4 inhibition and sargramostim reported an acceptable ORR and toxicity profile in this high-risk patient population (41% with CNS metastases) and with ipilumumab (3 mg/kg/dose) [101], which is lower than the Hodi et al. [99] and Kwek et al. [100] trials (ipilimumab 10 mg/kg/dose) (Table 4). These studies support the benefit of adding sargramostim to immune checkpoint blockade, which may result in improved disease control rates and lower rates of irAEs.

Immune checkpoint inhibitors are frequently combined for the treatment of malignancies as this approach provides an improved benefit but with a greater risk of toxicity. In melanoma, the combination of CTLA-4 and PD-1 inhibition has a higher efficacy than either therapy alone but is associated with a 55% rate of severe irAEs [106]. A clinical trial exploring the addition of sargramostim to CTLA-4 inhibition combined with PD-1 inhibition in melanoma is ongoing (ECOG-ACRIN6141; NCT02339571) [102] (Table 4). The phase 2 portion of this investigation demonstrated the safety of the triple combination which enabled the trial to move forward to phase 3.

Table 4 details other investigations of sargramostim with ICI therapies that have been completed for biliary cancer [103] and prostate cancer [104], and is ongoing for non-small cell lung cancer [105].

The effects of sargramostim on the immune infiltrates observed within tumors and its effect upon anti-cancer immunological activity remain unknown. One prior observation was that GM-CSF increased CD4+ and CD8+ ICOS T cells [99,107]. The presence of these cells has previously been correlated with a positive response to immune checkpoint blockade by Carthon et al. [108]. However, additional work is needed to understand these effects further.

## 5. Discussion

Many cancer patients are currently deriving significant benefits from the use of ICIs [1,2,3,4,5,6]. Unfortunately, these agents may cause irAEs that can be fatal, and the treatment of these irAEs can interfere with anti-cancer responses. We reviewed the underlying mechanisms associated with the development of GI irAEs. We further described the common symptoms and signs of GI irAEs and shared the lessons learned about GI irAEs from IBD.

GI irAEs often require endoscopy to properly manage as the extent of luminal involvement and severity of injury correlate poorly with symptoms and cross-sectional imaging [18,20]. Currently, the treatment for GI irAEs begins after the onset of symptoms using broad immune suppression; while generally effective, this approach runs the risk of inhibiting optimal anti-tumor responses. In particular, systemic glucocorticoids and TNF inhibitors may reduce the full benefit of immunotherapy [109,110]. A number of investigators have recognized the similarities between GI irAEs and IBD. As a result, the initial treatment strategies for GI irAEs mirror those used as standard therapies for IBD. The use of data in this fashion can represent a significant limitation, and currently there are no randomized controlled trials of a specific therapy for GI irAEs. We indicate that it is imperative to identify alternative, less immunosuppressive strategies for attenuating or preventing GI irAEs with the potential to improve oncologic outcomes. These approaches, unfortunately, are based on retrospective analyses or small patient populations. While first-line treatment for GI irAEs remains systemic glucocorticoid therapy, which is only effective in about two-thirds of patients, those subjects who fail to respond adequately must receive potentially more toxic treatments such as the IL-23p40 inhibitor ustekinumab and the JAK inhibitor tofacitinib (Table 3) [19,33,34]. The approach of using IBD as a surrogate for treating GI irAE obviously has limitations.

It is imperative to identify alternative, less immunosuppressive strategies for attenuating or preventing GI irAEs with the potential to improve oncologic outcomes. The risk of inadvertently interfering with anti-tumor immunity is even more important to consider when trying to develop prophylactic therapies for GI irAEs as, by definition, preventative therapies subject some patients to treatment that they do not need as they might not develop luminal inflammation. The development of these specific approaches will undoubtedly take considerable time and effort.

Sargramostim (glycosylated, yeast-derived rhu GM-CSF) is an agent that has been used in clinical practice for more than 30 years and has a well-recognized safety profile with much therapeutic potential as a host-directed immunotherapy. Sargramostim has the potential to act through a mechanism that, to date, is fundamentally different from other treatments used to manage GI irAEs. In some settings, such as the melanoma tumor microenvironment, GM-CSF can enhance the formation of cross-presenting dendritic cells, improving the presentation of tumor antigens and increasing the quality of anti-tumor T cell responses; these effects can potentially work synergistically with checkpoint blockade. This scenario represents at least one interpretation of the positive early clinical data from combination studies of sargramostim and CTLA-4 blockade. The enhanced activation of cross-presenting dendritic cells may not be a general mechanism that applies to all tumors. However, GM-CSF also can increase the formation of MDSCs in some tumor types. These cells may also play a role in the protective responses exerted by GM-CSF in the inflamed colon, and an improved barrier defense may also be important in the observed decrease in GI irAE risk (Figure 1).

The clinical data available thus far suggest that sargramostim can improve anti-tumor responses in melanoma while also reducing the risk for GI irAEs. In the multicenter, Eastern Cooperative Group (ECOG) advanced malignant melanoma study reported by Hodi et al. [99], the addition of sargramostim to ipilimumab significantly lowered grade 3–5 adverse events compared to the ipilimumab-alone arm (Figure 4). In fact, not only were GI irAEs significantly lowered, but pulmonary adverse events also were reduced in the sargramostim arm. These are the first data to suggest the efficacy of prophylactic irAE treatment that does not interfere with anti-tumor responses. We do not know if this prophylactic effect in GI irAE management will hold in other therapeutic settings, or if the doses currently used as prophylaxis are therapeutically appropriate in this context. Clinical trials to determine how sargramostim affects the inflamed colon in GI irAEs will be an important next step and could have a substantial impact on the standard management of this important checkpoint blockade toxicity.

## 6. Conclusions

While ICI therapy can provide significant anti-cancer responses in many patients, irAEs can accompany this therapy and lead to significant morbidity. In particular, the GI tract is a major organ affected by ICI therapy. GI irAEs are common and can be severe or even fatal. Further, the treatment for GI irAEs may impair anti-cancer responses. Thus, we focused on this specific organ toxicity and addressed the prompt recognition, diagnosis, and treatments that differ according to extent of injury (i.e., mild and moderate/severe). As the treatment for GI irAE actually may impair anti-cancer responses, we discussed both the rationale and evidence for the use of sargramostim as a treatment. Sargramostim has a well-recognized safety profile and appears, in preclinical and clinical studies, to have a unique mechanism of action as a host-directed immunotherapy (Figure 3) that can treat irAEs and yet not impair the anti-cancer effects of other agents (Table 4). As such, we suggest further investigations of irAE as a prophylaxis. It is important to note that much of the literature thus far on the combination of sargramostim with ICIs is in the context of malignant melanoma treatment and the development of irAEs while patients are receiving treatment. While it is clear the underlying biology of irAEs, particularly GI irAEs arising from dysregulated barrier function, is not expected to differ on the basis of primary malignancy, prospective trial data in other cancers are urgently needed. Further, whether other determinants of checkpoint blockade efficacy and safety—such as the gut microbiome or combination therapy—may influence this process remains to be seen. Other strategies to prevent or ameliorate the morbidity and costly complications of ICI therapy that do not blunt the immune system’s anti-tumor response remain an active area of investigation.

## Figures and Tables

**Table 1:** Symptoms and frequency of immune checkpoint inhibitor-induced enterocolitis.

Grading	Symptoms	CTLA-4 Inhibitor Frequency (%)	PD-1/PD-L1 Inhibitor Frequency (%)
Mild	Diarrhea, nausea, vomiting, decreased appetite, reflux	20–40	7–20
Moderate/Severe	Diarrhea (commonly watery, can be bloody), nausea, vomiting, decreased appetite, reflux, cramping, urgency, fever	15–20	2–5

**Table 2:** Comparison of immune checkpoint inhibitor-induced gastrointestinal immune-related adverse event features with inflammatory bowel disease (i.e., ulcerative colitis and Crohn’s disease).

Features	ICI GI irAEs	Ulcerative Colitis	Crohn’s Disease
Symptoms	Nausea and vomiting **(more often than with UC and CD)** Urgency, cramping, and frequent watery bowel movements Abdominal pain Bloody diarrhea **(less frequent than with UC or CD)** **Rapid escalation**	Nausea and vomiting Urgency, cramping, and frequent watery bowel movements Abdominal pain Bloody diarrhea **Weight loss**	Nausea and vomiting Urgency, cramping, and frequent watery bowel movements Abdominal pain Bloody diarrhea **Weight loss** **Obstruction** **Fistulas**
Location	Continuous distribution starting in the rectum and **most often involving the entire colon** **Rare colonic patches of “skip lesions”** **Stomach or small bowel sometimes affected**	Continuous distribution starting in the rectum and only **involving the colon**	**Distinct patches or “skip lesions” that can involve the entire GI tract**
Morphological Changes	Edema Erythema Spontaneous bleeding Erosions and deep ulcerations (occasionally)	Edema Erythema Spontaneous bleeding Erosions and deep ulcerations (occasionally)	Edema Erythema Spontaneous bleeding Erosions and deep ulcerations **(more common than in UC/ICI colitis)** **Strictures** **Fistula**
Biopsy Results	**Lymphocytic and neutrophilic inflammation** **No evidence of chronic changes**	**More often neutrophilic inflammation** **Evidence of chronic changes**	**More often neutrophilic inflammation** **Evidence of chronic changes**

Note: Considerable overlap exists for the features in ICI GI irAEs and ulcerative colitis and Crohn’s disease; bold text highlights the differences between conditions. Abbreviations: CD = Crohn’s disease; GI = gastrointestinal; ICI = immune checkpoint inhibitor; irAE = immune-related adverse event; UC = ulcerative colitis.

**Table 3:** Common treatments for immune checkpoint inhibitor enterocolitis based on inflammation grading.

Stage of Rescue Therapy	Mild	Moderate/Severe
First-line	Systemic glucocorticosteroid (oral; IV if refractory)	Systemic glucocorticosteroid (IV) ± TNF-alpha inhibitor (e.g., infliximab) OR integrin inhibitor (e.g., vedolizumab)
Second-line	TNF-alpha inhibitor (e.g., infliximab) OR integrin inhibitor (e.g., vedolizumab)	If not responding to TNF-alpha inhibitor (e.g., infliximab) OR integrin inhibitor (e.g., vedolizumab), switch treatment class
Third-line	IL-23p40 inhibitor (e.g., ustekinumab) OR JAK inhibitor (e.g., tofacitinib)	

Abbreviations: IV = intravenous; JAK = Janus kinase; TNF = tumor necrosis factor.

**Table 4:** Clinical studies of sargramostim with immune checkpoint inhibitors.

Citation	Patient Population and Study Design	Treatment	Efficacy	Adverse Events	Comments
Melanoma					
Hodi 2014 (ECOG 1608) [99]	Melanoma (unresectable stage III/IV; N = 245); Phase 2, randomized	Ipilimumab 10 mg/kg IV d1 + sargramostim 250 μg SC d1-14 of 21 d cycles × 4, then maintenance vs. ipilimumab alone	*Ipilimumab +* *sargramostim vs.* *ipilimumab alone:* Median OS 17.5 m vs. 12.7 m (*p* = 0.01) Median PFS 3.1 m vs. 3.1 m (*p* = NS) Disease control ^1^ (median follow up 13.3 m) 36.6% vs. 33.6% ORR ^1^ (median follow up 13.3 m) 15.5% vs. 14.8% (*p* = NS)	*Ipilimumab +* *sargramostim vs. ipilimumab* *alone:* Grade 3–5 overall TRAEs 45% vs. 58% (*p* = 0.04) Grade 3–5 GI TRAEs 16% vs. 27% (*p* = 0.05) Grade 3–5 pulmonary TRAEs 0% vs. 7.5% (*p* = 0.003) Grade 3–5 treatment-related colonic perforation 1.7% vs. 5.8%	Increased OS with sargramostim Decreased grade 3–5 overall, GI and pulmonary AEs, and colonic perforation with sargramostim
Kwek 2016 [100]	Melanoma (unresectable stage III/IV; N = 22); Phase 2, single-arm	Ipilimumab 10 mg/kg IV d1 + sargramostim 125 µg/m^2^ SC d1-14 of 21 d cycles × 4, then sargramostim × 3 m, then ipilimumab + sargramostim maintenance	Median OS 21.1 m Median PFS 3.5 m Disease control ^2^ (24 w) 41% ORR ^2^ (24 w) 32%	Grade 3–4 overall AEs 41% No deaths	OS and Grade 3–5 AEs similar to randomized trial
Luke 2015 [101]	Melanoma (metastatic (41% with CNS metastases); N = 32); Retrospective	Ipilimumab 3 mg/kg IV d1 + sargramostim 250 µg SC d1-14 of 21 d cycles × 4	Median OS 41 w Median TTP 13.7 w Disease control ^2^ (median follow-up 37 w) 44% ORR ^2^ (median follow-up 37 w) 20%	Grade 3–4 TRAEs 9.4% No deaths	Authors report an acceptable ORR in this poor-risk patient population and with currently approved ipilimumab dosing (lower than randomized trial) Favorable toxicity profile with sargramostim compared to historical data without sargramostim
EA6141/NCT02339571 (estimated completion 2033) [102]	Melanoma (unresectable stage III/IV); Phase 2, randomized	Ipilimumab 3 mg/kg IV d1 + nivolumab 1 mg/kg IV d1 + sargramostim 250 µg SC d1-14 of 21 d cycles × 4, then maintenance nivolumab + sargramostim vs. ipilimumab + nivolumab, then nivolumab alone	Ongoing	Ongoing	Phase 2 portion met safety requirements; phase 3 is ongoing
Biliary Cancer					
Kelley 2022 (abstract only available) [103]	Advanced biliary cancer (N = 42); Phase 2, single-arm	Pembrolizumab 200 mg IV d1 + sargramostim 250 µg SC d1-14 × 2 of 3 21 d cycles	Median OS 393 d Median PFS 63 d ORR ^1^ 12%	Grade 3–4 TRAE 10% Serious TRAEs 7%	Authors report safety and tolerability of sargramostim with pembrolizumab Authors report ORR with sargramostim + pembrolizumab higher than expected with pembrolizumab monotherapy
Prostate Cancer					
Kwek 2015 [104]	Prostate cancer (metastatic, castration-resistant; N = 42); Phase 1b, dose-escalation study	Ipilimumab escalating doses 0.5–10 mg/kg IV d1 + sargramostim 250 µg/m^2^ SC d1-14 of 28 d cycles × 4	Median OS 23.6 m Median TTP 20 m	Dose-limiting toxicities 4.8%	Durable benefit and long-term survival observed Toxicity similar to other ipilimumab studies
Non-Small Cell Lung Cancer					
NCT04856176 (estimated completion 2024) [105]	Non-small cell lung cancer (stage III/IV); Phase 2, single-arm	Chemo-immunotherapy × 4 cycles, then pembrolizumab + sargramostim ± pemetrexed	Ongoing	Ongoing	Ongoing

^1^ Response measured by Response Evaluation Criteria in Solid Tumors (RECIST) v1.1; ^2^ response measured by Immune-Related Response Criteria (irRC). Abbreviations: AE = adverse event; CNS = central nervous system; d = day; EA = ECOG-ACRIN; ECOG = Eastern Cooperative Oncology Group; GI = gastrointestinal; IV = intravenous; m = month; NS = not significant; ORR = overall response rate; OS = overall survival; PFS = progression-free survival; SC = subcutaneous; TRAE = treatment-related adverse event; TTP = time to progression; w = week.
